# MetoksyKval: the extent of pre-hospital methoxyflurane administration for acute traumatic pain: focus on economic impact and rationale for use

**DOI:** 10.1186/s13049-026-01546-z

**Published:** 2026-01-09

**Authors:** Randi Simensen, Live Smalberget, Fridtjof Heyerdahl

**Affiliations:** 1 Institute of Clinical Medicine, University of Oslo, Oslo, Norway; 2 Department of Research and Development, Norwegian Air Ambulance Foundation, Oslo, Norway; 3 Pre-Hospital Division, Innlandet Hospital Trust, Moelv, Norway; 4 Division of Pre-Hospital Services, Oslo University Hospital, Oslo, Norway

**Keywords:** Methoxyflurane, Trauma pain, Pre-hospital emergency care, Health economics, Inhaled analgesic, Ground ambulance service

## Abstract

**Background:**

Effective pain management is critical in pre-hospital care, yet many patients still receive suboptimal treatment. Methoxyflurane, a handheld self-administered analgesic, is increasingly used in European ambulance services. Its effectiveness and safety are established, but the reasons for its use in acute pre-hospital settings have received limited attention. This study aimed to explore patterns of use, effectiveness, and economic implications of methoxyflurane in traumatic pain, with particular focus on decision-making and patient satisfaction.

**Methods:**

This prospective observational study in a Norwegian ground ambulance service, conducted from January 15 to July 15, 2024, involved adult patients (aged ≥ 18 years) with moderate to severe traumatic pain treated by the ground ambulance service of Innlandet Hospital Trust. Methoxyflurane was administered at the discretion of the ambulance personnel. Pain intensity scores, demographic characteristics, treatment satisfaction levels, and rationale behind choosing methoxyflurane were recorded.

**Results:**

A total of 48 patients were included (median age 69 years, 58% male), most with fall-related injuries (77%) and fractures (65%). In the corresponding 2022 period, only 41% of patients with an Numeric rating Scale (NRS) pain score ≥ 4 received any analgesia. In this study, methoxyflurane accounted for 7% of such cases but increased overall analgesic costs three-fold. It was selected in 15/48 (31%) cases due to difficult intravenous access, in 15/48 (31%) as a preferred non-opioid option despite NRS ≥ 4, and in 13/48 (27%) to reduce on-scene time. Pain measured on NRS decreased from a median of 8 at baseline to 5 within ten minutes, with 52% achieving sufficient relief without rescue medication. Satisfaction was good or better in 61%, 67% would choose methoxyflurane again, and 90% of personnel reported a strong preference for future use. Adverse events were mild and comparable to earlier studies.

**Conclusions:**

Methoxyflurane was viewed as a useful non-intravenous option in settings where rapid analgesia is essential. Although used in only 7% of patients with moderate to severe pain and associated with higher costs, it provided fast pain relief in patients with difficult venous access and when minimising on-scene time was critical. These results support its role as a bridge to longer-acting intravenous analgesia and may help decision-makers identify patient groups most likely to benefit, enabling more targeted and cost-effective implementation.

**Trial registration:**

Approved by the Regional Ethics Committee, South East; https://rekportalen.no, ref no. 658708/ 255159 and Data Protection Officer at Innlandet Hospital Trust ref.no 28028904).

**Supplementary Information:**

The online version contains supplementary material available at 10.1186/s13049-026-01546-z.

## Background

Oligoanalgesia, defined as inadequate pain treatment [1] remains a persistent problem in the pre-hospital setting [2] despite long-standing awareness [3–5]. While intravenous opioids are widely regarded as the gold standard, safety concerns and difficulties establishing intravenous access have maintained interest in rapid-onset alternatives [6, 7]. Despite established guidelines, pain management in ambulance services still falls short, as more than 70% of patients arrive at hospital in pain [8] and up to 53% experience inadequate pain relief [9, 10]. These observations illustrate an ongoing gap between recommended practice and clinical reality. Such limitations are not trivial, as inadequate treatment may worsen short-term outcomes and increase the risk of chronic pain [11]. Thus, identifying effective and practical treatment strategies remains central to optimising pre-hospital care.

Methoxyflurane was introduced into the European pre-hospital setting in 2015 [12], and represents a valuable expansion of analgesic options available to ambulance personnel. Its proposed advantages include self-administration; this may increase patient involvement. Real-world experiences from ambulance [13] and ski patrols [14] suggest its effectiveness. At the same time, the higher cost (€34.78 per dose compared to €3.70 for a morphine ampoule, Additional file 1) and the possible higher need for additional analgesics, raise questions about methoxyflurane’s role in practice. Findings from our PreMeFen randomised controlled trial, showed that methoxyflurane was non-inferior to intranasal fentanyl and intravenous morphine, with early advantages, while intranasal fentanyl was less effective than morphine the first ten minutes after administration [15]. This comparison does not account for the additional time required to establish intravenous access prior to morphine delivery, which delays the onset of analgesia in clinical practice.

The need for non-intravenous analgesia is clear, and methoxyflurane has been discussed as a possible bridge to longer-acting intravenous agents [16–18]. Together, these findings add to the ongoing conversation about whether methoxyflurane should be considered for initial use in pre-hospital care. Limited attention has been paid to when and how ambulance personnel decide to administer methoxyflurane, and how patient characteristics, situational factors, and the availability of other analgesics influence these decisions. This study explores patterns of use, effectiveness, and economic implications in the treatment of traumatic pain, with particular focus on personnel decision-making and patient satisfaction.

The overarching objectives of this study are as follows.

### Primary objectives

To determine the frequency and extent of methoxyflurane administration in the pre-hospital setting with a focus on economic impact, and to assess the rationale for its use by ambulance personnel.

### Secondary objective

To assess the effectiveness, safety, side effects, and user satisfaction of methoxyflurane use with established evidence.

## Methods

### Design and setting

MetoksyKval was a prospective observational study from 15 January to 15 July 2024 in the ground ambulance service of Innlandet Hospital Trust (IHT), Norway’s third-largest, serving 360,000 residents across rural and urban areas [19]. Patients were recruited from the Gran, Gjøvik, and Lillehammer ambulance stations, the same sites as the PreMeFen trial (see map Additional file 2). Each ambulance had two crew members [20] who were either Emergency Medical Technicians, paramedics, or nurses. Before enrolment, 51 ambulance personnel completed mandatory online training to standardise data collection and ensure adherence to the study protocol. Study personnel coverage was defined as at least one trained personnel per ambulance, which was calculated to cover 85% of all missions (Additional file 3). The STROBE checklist guided study reporting [21] (Additional file 4).

### Inclusion criteria

Methoxyflurane was integrated into the standard analgesia protocol, with use left to the discretion of ambulance personnel. Eligible patients, according to product characteristics [22], were adults (≥ 18 years) with traumatic pain, Glasgow Coma Scale 15, and self-reported pain ≥ 4 on a 0–10 pain Numeric Rating Scale (NRS). Exclusion criteria were patients younger than 18 years, Glasgow Coma Scale < 15 or clinical conditions contraindicating methoxyflurane (Additional file 5). On-site informed consent was obtained after ambulance personnel read a predefined text; oral consent was witnessed and documented by both ambulance personnel (Additional file 6). All patients received written study information, including withdrawal procedures, in accordance with Norwegian regulations.

### Data collection

Data were obtained from ambulance records and a study-specific case report form, covering demographics, injury site, causal factors, diagnosis, intravenous access, weather conditions, and all analgesic treatments, including additional pain medication. Pain intensity was measured with the 0–10 NRS at baseline, 5 min, 10 min, and at handover; severe pain was defined as NRS 7–10. Vital signs (oxygen saturation, pulse) were recorded at baseline and handover. Patient benefit was assessed using a 5-point Likert scale, and participants’ willingness to receive methoxyflurane in a similar situation was recorded. Adverse events (AE) were documented using predefined categories and a free-text field, coded by the study team. Ambulance personnel assessed operational benefit. Missing pain NRS values were not imputed; results are presented with observed denominators. Sex was determined from the national ID number. Data were entered in Excel, double-checked by the study team, and stored at IHT in line with data protection regulations. Comparative data from the same period in 2022 were retrospectively extracted from ambulance records and adjusted for 85% study personnel coverage.

### Treatment procedure

Methoxyflurane administration followed routine clinical practice. Before treatment, Pain NRS was patient-reported and vital signs were measured with the LifePak 15 monitor (Stryker, US). Patients self-administered methoxyflurane (Penthrox®, Medical Developments NED B.V.) after instruction from ambulance personnel. The maximum dose was two ampules (6 ml). If pain relief was insufficient, ambulance personnel followed the health trust’s standard analgesic protocols (Additional file 7).

### Statistical analysis

Statistical analysis was conducted using data from the 48 patients included in the full analysis set. A separate safety analysis involved 50 participants, including two patients who received methoxyflurane, although they did not meet the inclusion criteria. Power calculations were not performed, as the number of inclusions was defined as an outcome itself. Categorical variables are presented as counts and percentages, which allows for a clear depiction of frequency within the study group. Continuous variables are summarised using univariate statistical measures, including medians and interquartile ranges (IQR) or means and standard deviations where appropriate. Missing values for the pain NRS are not imputed in the analysis, and detailed information about missing pain NRS values is provided in Additional file 8. Analyses were conducted using STATA version 19 (StataCorp LLC, College Station, TX, USA). Aggregated data on pain NRS scores and analgesic treatment from the six months of 2022 are summarised as frequencies and percentages to provide insights.

## Results

### Participant characteristics and incident trends

**Fig. 1:**
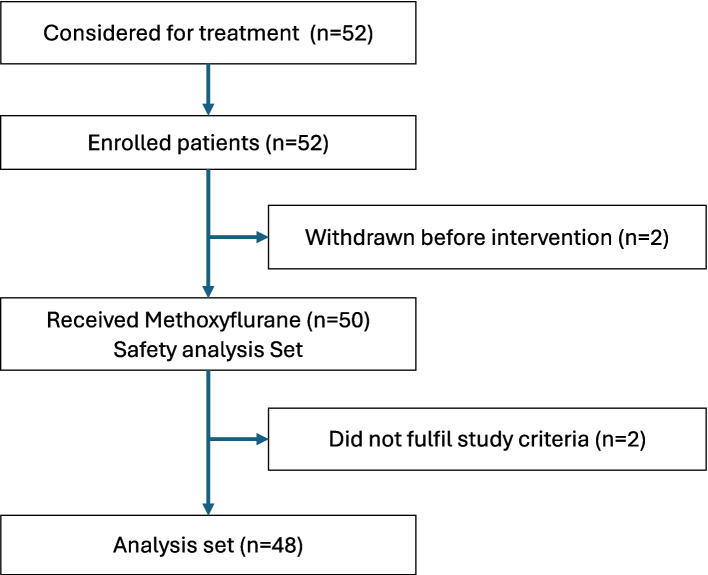
Flowchart of patient inclusion and analysis set Flow diagram of study enrolment. Two patients were excluded due to not fulfilling the inclusion criteria (under 18 years of age)

Between January 15 and July 15, 2024, 52 patients were considered for treatment; two withdrew before administration, leaving 50 who received treatment, and were included in the safety set. Two patients under the age of 18 years were erroneously included, and hence the full analysis set comprised of 48 patients (Fig. 1).

**Fig. 2:**
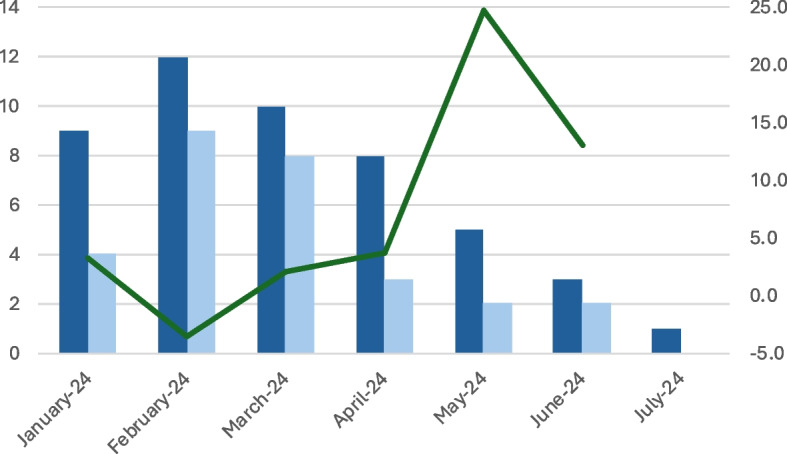
Inclusion rates, outdoor cases, and mean temperature. Legend: The figure shows seasonal variation in patient inclusion. Dark blue bars represent monthly inclusions, light blue bars outdoor cases, and the green line mean monthly temperature in Celsius (°C)

Of these, 28/48 (58%) were male and the median age was 69 years (range 18–91). Most incidents occurred at home (26/48, 54%), and outdoors (25/48, 52%) (Table 1, Fig. 2). Falls were the most common mechanism of injury (37/48, 77%) and fractures the most frequent tentative diagnosis (31/48, 65%).

**Table 1:** Demographic

	**Overall, *N* = 48**
**Sex**	**n (%)**
Male	28 (58%)
Female	20 (42%)
**Age (Year)**	**n (Range)**
Median	68.5 (18–91)
**Tentative Diagnosis**	**n (%)**
Fracture	31 (65%)
Miscellaneous	6 (13%)
Blunt soft tissue injury	5 (10%)
Luxation	5 (10%)
Cuts	1 (2%)
**Place of injury**	**n (%)**
Indoors (home)	14 (29%)
Outdoors (home)	12 (25%)
Outdoors (nature/sea)	7 (15%)
Outdoors (public)	6 (13%)
Indoors (Public)	5 (10%)
Miscellaneous	4 (8%)
**Casual Factors**	**n (%)**
Fall-injury	37 (77%)
Miscellaneous	8 (17%)
Crush-Injury	2 (4%)
Transport accident	1 (2%)
**Handover**	**n (%)**
Handover at Emergency Department	39 (81%)
Handover at outpatient clinic	8 (17%)
Left on scene	1 (2%)

Legend: Values are presented as number (percentage) unless otherwise indicated. Age is given as median (range)

Methoxyflurane alone provided sufficient pain relief in 25/48 (52%), of which 6/48 (13%) required a second dose. Additional pain relief medication was administered to 23/48 (48%), most often opioid–paracetamol or opioid–esketamine combinations; details are provided in Table 2. Hence, Methoxyflurane replaced other analgesics in 25 cases, and a total of 54 doses methoxyflurane were administered.

**Table 2:** Additional analgesic administered

	**Number of Patients**	**% of N = 48**
No additional analgesic	25	52%
Received additional analgesic	23	48%
Opioid + Paracetamol	5	10%
Opioid + Esketamine	5	10%
Paracetamol	4	8%
Opioid	3	6%
Esketamine	2	4%
Esketamine + NSAID	2	4%
Opioid + Paracetamol + NSAID	2	4%

*NSAID* nonsteroidal anti-inflammatory drug

Percentages are calculated from the total number of patients (N = 48)

During a corresponding six-month period in 2022 (from the same three ambulance stations and adjusted for 85% study personnel coverage), 1206 patients aged > 18 years had a pain score NRS (0–10) documented in their electronic patient journal. Of these, 677 patients had pain NRS > 4 and 277/677 (41%) received analgesic treatment (see Table 3). The extent of methoxyflurane use in MetoxyKval would hence represent 48/677 (7%) of all patients with NRS > 4, and 48/277 (17%) of patients receiving any analgesic treatment.

**Table 3:** Retrospective 2022 Comparison Group: Patient Characteristics and Analgesic Strategies

	**Number of Patients**	**% of N = 677**
Overall Ambulance assignment^*^	8103	
Age > 18 and Pain NRS 0–10	1206	
Age > 18 and Pain NRS 0–3	904	
Age > 18 and Pain NRS ≥ 4	677	100%
**Sex**		
Female	359	53%
Male	319	47%
**Received analgesic treatment**	277	41%

Ledged: Comparative data from 2022 were retrospectively extracted from ambulance records and adjusted for 85% study personnel coverage

^*^All mission types, including hospital transport and on-scene treatment

Based on the current pricing, the cost of analgesics administered during the 2022 period was €3.7 per patient receiving analgesics and €1,5 per patient with a pain NRS score greater than 4. The cost of cannulas, syringes etc. is not included in the estimates. The current cost per dose of methoxyflurane is €34.8. Replacing other analgesics in 25 cases, methoxyflurane adds to the total costs of analgesics from € 925 to € 2804, a threefold increase. Hence, an increase in the cost per patients receiving analgesics from € 3,7 to € 10,1, and an increase in cost per patients with NRS scores > 4 from € 1.5 to € 4.1.

### Rationale for choosing methoxyflurane

**Fig. 3:**
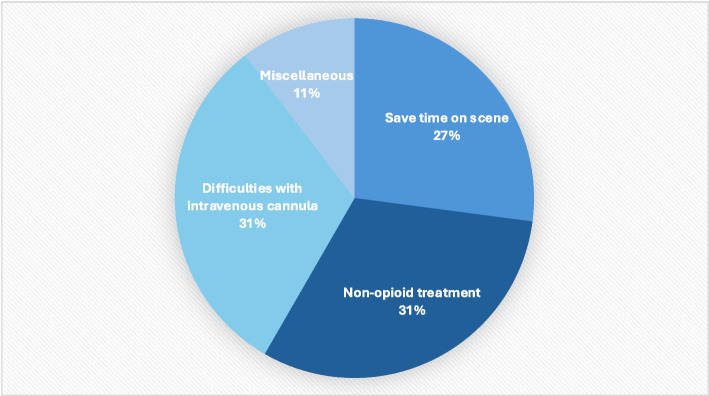
Reasons for methoxyflurane use. Legends: Reported reasons for administering methoxyflurane

Methoxyflurane was chosen in 15/48 (31%) cases because of difficulty establishing intravenous access, in 15/48 (31%) as a preferable non-opioid alternative despite pain NRS ≥ 4, in 13/48 (27%) to save time, and in 5/48 (11%) for other reasons. (Fig. 3). Intravenous cannulation was established in 29/48 (60%). The most common reasons for not establishing venous access was failed attempts, (Additional file 9).

### Effectiveness

At baseline, median pain NRS score was 8 (IQR 6–10) and 34/48 (71%) reported severe pain (NRS 7–10). Pain intensity decreased to median NRS 5 at five minutes (IQR 4–8; one missing) and remained on five at ten minutes (IQR 4–7; four missing). The overall pain NRS change from baseline to handover was −3 points (IQR –2 to –5), and 29/48 (60%) experienced a reduction ≥ 2 points within ten minutes.

Median time from ambulance arrival to first vital-sign assessment was nine minutes (IQR 5–15); the first methoxyflurane dose was given a median two minutes later (IQR 1–5).

**Fig. 4:**
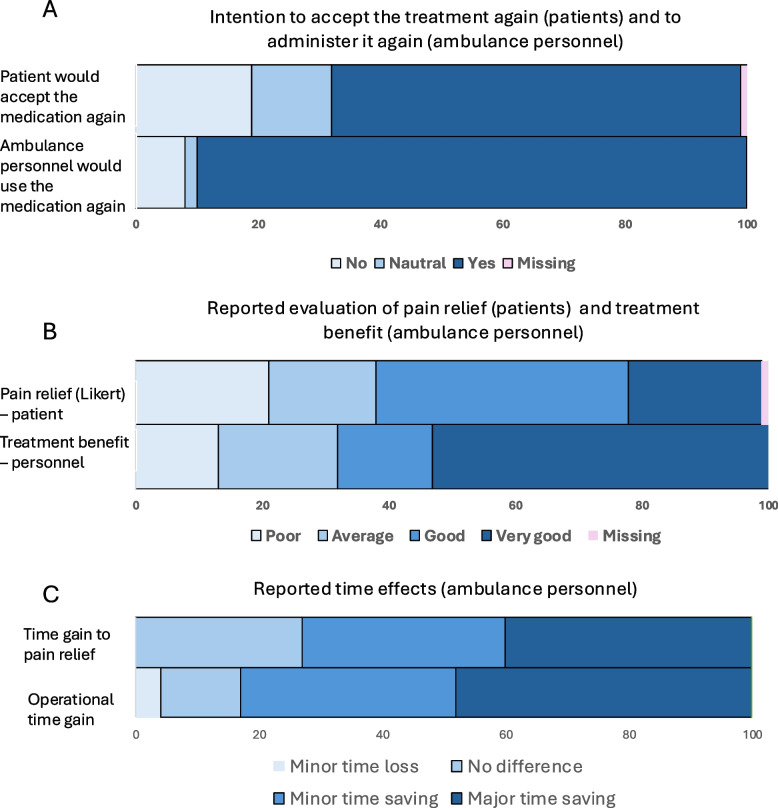
Patient- and ambulance personnel–reported outcomes. Legends: Panel **A**: Comparison of intention to accept the treatment again (patients) and to administer it again (ambulance personnel). Panel **B**: Comparison of patient-rated pain relief and ambulance personnel-perceived treatment benefit. Panel **C**: Comparison of reported time effects — time to pain relief and operational efficiency

Among patient-reported outcomes, 29/47 (61%) rated pain relief as good or very good, while 10/47 (21%) reported poor or very poor relief. In the same group 32/47 (67%), indicate they would be willing to use the treatment again, a view supported by ambulance personnel, of whom 43/48 (90%) indicated they would apply the same methoxyflurane strategy again in a similar context. Perceived treatment benefit as assessed by ambulance personnel after each mission was generally high (33/48, 69%). A time-saving effect was most evident for time to pain relief (23/48, 48%), whereas fewer ambulance personnel reported gains in operational efficiency (19/48, 40% (see Fig. 4).

### Assessment of safety

Safety analysis included 50 patients. In total, 43 AEs occurred in 33/50 (66%); 10/50 (20%) experienced more than one AE. The most frequent AEs were euphoria 17/50 (34%), drowsiness 13/50 (26%), and dizziness 6/50 (12%) (Table 4).

**Table 4:** Adverse events

Patients with adverse events	n	%
Patients with AE	33	66%
Patient without AE	17	34%
**Total**	**50**	**100%**
** Types of adverse event**	**n**	**%**
Euphoria	17	40%
Drowsiness	13	30%
Dizziness	6	14%
Nausea	3	7%
Coughing	3	7%
Headache	1	2%
**Total AEs reported**	**43**	**100%**

*AE* adverse event

Number of adverse events registered as the second adverse event, in total 10 patients. Percentages for adverse event types are calculated from the total number of adverse events (n = 43)

## Discussion

This prospective observational study evaluated the use, rationale and effectiveness of methoxyflurane in traumatic pain among adult patients in the ground ambulance services. Methoxyflurane was used in 17% of all patients receiving analgesic treatment, resulting in an increase in analgesia-related costs of 6.4 € per treated patient with analgesics. Methoxyflurane was primarily chosen as a non-opioid alternative to intravenous morphine; when intravenous access was difficult; and to save time in the pre-hospital setting. Methoxyflurane was perceived as a valuable non-intravenous alternative among patients and caregivers. Because introduction of methoxyflurane increase overall analgesic costs, identification of patients who benefits the most of this non-intravenous alternative is important.

### Interpretation of findings

Methoxyflurane was selected in 15/48 (31%) of cases because ambulance personnel considered non-opioid treatment sufficient, despite baseline pain NRS ≥ 4. One plausible explanation for this decision is that although methoxyflurane pharmacologically is comparable to paracetamol [17], it may be perceived by ambulance personnel as more potent. The rapid onset, inhalational route, and patient-controlled administration may contribute to its use as an alternative to opioids or ketamine, particularly when concerns related to opioid exposure, monitoring requirements, or escalation of care are present [23]. This decision-making aligns with Häsle et al. [24], who reported discrepancies between patient and paramedic pain assessments. Almost half of the patients 23/48 (48%) required rescue medication. However, methoxyflurane provided clinically relevant relief for most patients, and because the median baseline pain was high (NRS 8), a high proportion of rescue medication should be expected. These observations are consistent with larger randomised controlled trials reporting rapid onset and effective pain reduction [16, 25–27]. Pain intensity decreased by a median of three NRS points from baseline to handover.

The inclusion of patient and use of methoxyflurane was highest in the early stage of this study, likely reflecting early enthusiasm, but stabilised over time. Another reason for declining inclusion rate is that ambient outdoor temperature increases from winter to summer (Fig. 2). Difficulties with intravenous access is higher during cold seasons outdoor. Hence, one can expect a higher use of methoxyflurane during winter season compared to summer.

The additional cost in this study is compared to the costs of conventional analgesics. As only 41% of patients with NRS > 4 received any analgesic treatment, oligoanalgesia seems evident. If all the patients with NRS > 4 received analgesia, the increase in cost per patient introducing methoxyflurane would have been less than twofold, instead of threefold. In addition, increase in cost should be considered against its clinical value. Although the present material cannot establish a definitive cost–benefit, methoxyflurane appears to offer a clear benefit for selected patients with rapid onset of pain relief in settings where intravenous analgesics take more time or is difficult to establish. Targeted protocols may best guide wider implementation to ensure it is reserved for those most likely to benefit. This study was not designed to compare methoxyflurane with other non-intravenous analgesic alternatives. Although the PreMeFen trial demonstrated that intranasal fentanyl was less effective (ref), other non-intravenous options may offer advantages, including lower costs. Future research should therefore also address the cost-effectiveness of alternative rapid-onset agents, such as intranasal opioids or ketamine, as well as other inhaled analgesics, evaluated within real-world, routine clinical settings [28, 29].

### Operational aspect

From an operational perspective, the median time from ambulance arrival to first dose was about 15 min**,** reflecting the necessary interval for vital assessments and clinical evaluation. This underlines the real-world complexity of pre-hospital care and demonstrates how timely, non-intravenous options can be integrated. Half of the patients required rescue medication, often in combination with other medicines. While paracetamol remains the cornerstone of the World Health Organisation pain ladder [30], acute pre-hospital care usually prioritises fast-acting agents. In this context, methoxyflurane can serve as a bridge, complementing standard protocols and supporting tailored analgesia. Self-administration and control with the methoxyflurane inhaler may explain high satisfaction, with 34/48 (71%) willing to use it again. This finding is in line with prior reports of good acceptability in real-world settings such as ski patrols [14, 31]. Although equipotency studies suggest relatively low analgesic efficacy [32], patient-reported outcomes emphasise its practical and psychological value. Factors such as anxiety, context and perceived control may influence both pain and treatment effect [33]. Our findings indicate that pain management remains suboptimal, suggesting that accessible, non-opioid alternatives could address unmet needs. Although methoxyflurane is more costly than opioids, potential time saved on scene and potential prevention of inadequate analgesia justify further health-economic evaluation [34].

### Assessment of safety

AE were reported in 33/50 (66%) patients, most commonly euphoria, drowsiness, and dizziness. This incidence was higher than in earlier studies [10, 35, 36], and may reflect our prospective and systematic reporting approach rather than retrospective data [37]. All AE were mild and transient, consistent with previous evidence of safety in pre-hospital use.

### Strengths and limitations

This study provides a real-world evaluation of methoxyflurane, assessing not only pain intensity and effectiveness but also operational aspects, safety, and ambulance personnel decision-making. The prospective design with predefined analyses and systematic data collection during treatment and enroute ensured robust data quality. Inclusion of both patient-reported outcomes and personnel assessments further strengthens the relevance of the findings to routine pre-hospital clinical practice**.** However, such an observational study performed in an everyday emergency setting, might be subject to potential selection and information bias. Inclusion depended on personnel judgement, and documentation occurred during acute care. The study was conducted in selected regions of a single ambulance service, which may limit generalisability, although the catchment area included both urban and rural populations comparable to other European settings. No formal power calculation was performed, as the number of inclusions was defined as an outcome of the study itself. The high utilisation observed early in the study may reflect novelty effects and increased awareness at initiation. Subjective evaluations by ambulance personnel could introduce reporting bias, particularly regarding treatment rationale and operational benefits. Nevertheless, triangulation with patient-reported outcomes adds robustness to the findings. Comparative analyses with 2022 data should be interpreted cautiously, as patient populations and clinical practice may have differed. Conducting the study in the same regions as the PreMeFen trial (2021–2023) may have also shaped attitudes toward methoxyflurane and affected external validity. Finally, although cost considerations are important, the study was not designed to assess cost-effectiveness for broader implementation.

## Conclusion

Methoxyflurane was perceived as a valuable non‑intravenous option for situations in which timely pain relief is critical. Its use accounted for 7% of all patients with moderate to severe pain and was associated with increased costs. Methoxyflurane provided rapid analgesia in patients with difficult venous access and in circumstances where reducing on‑scene time is important, supporting its role as a bridge to longer‑acting intravenous analgesia. These findings can help decision‑makers identify patient groups most likely to benefit and guide targeted implementation to achieve a favourable cost‑effectiveness balance.

## Supplementary Information

Additional file 1: Cost overview of analgesics.

Additional file 2: Location at ambulance station including in the MetoksyKval study.

Additional file 3: Calculation: Probability of at Least One Trained Crew Member.

Additional file 4: STROBE cheklist.

Additional file 5: Inclusion and exclusion criteria.

Additional file 6: Translated version of Consent form.

Additional file 7: Additional treatment per Innlandet Hospital Trust analgesic protocols.

Additional file 8: Numeric Rating Scale of pain with missing values.

Additional file 9: Intravenous access and reported reasons for omission.

## Data Availability

Anonymised individual data and data dictionary will be available for research purposes by request to the corresponding author. Provided that ethical approval is granted.

## Abbreviations

AE: Adverse Event
IHT: Innlandet Hospital Trust
NRS: Numeric Rating Scale
IQR: Interquartile Range
