# Correction: MetoksyKval: the extent of pre‑hospital methoxyflurane administration for acute traumatic pain: focus on economic impact and rationale for use

**DOI:** 10.1186/s13049-026-01586-5

**Published:** 2026-03-18

**Authors:** Randi Simensen, Live Smalberget, Fridtjof Heyerdahl

**Affiliations:** 1 Institute of Clinical Medicine, University of Oslo, Oslo, Norway; 2 Department of Research and Development, Norwegian Air Ambulance Foundation, Oslo, Norway; 3 Pre‑Hospital Division, Innlandet Hospital Trust, Moelv, Norway; 4 Division of Pre‑Hospital Services, Oslo University Hospital, Oslo, Norway

**Correction: Scand J Trauma Resusc Emerg Med 34, 29 (2026)**

**https://doi.org/10.1186/s13049-026-01546-z**

Following publication of the original article [1], the authors would like to update figure 4 of the published article.
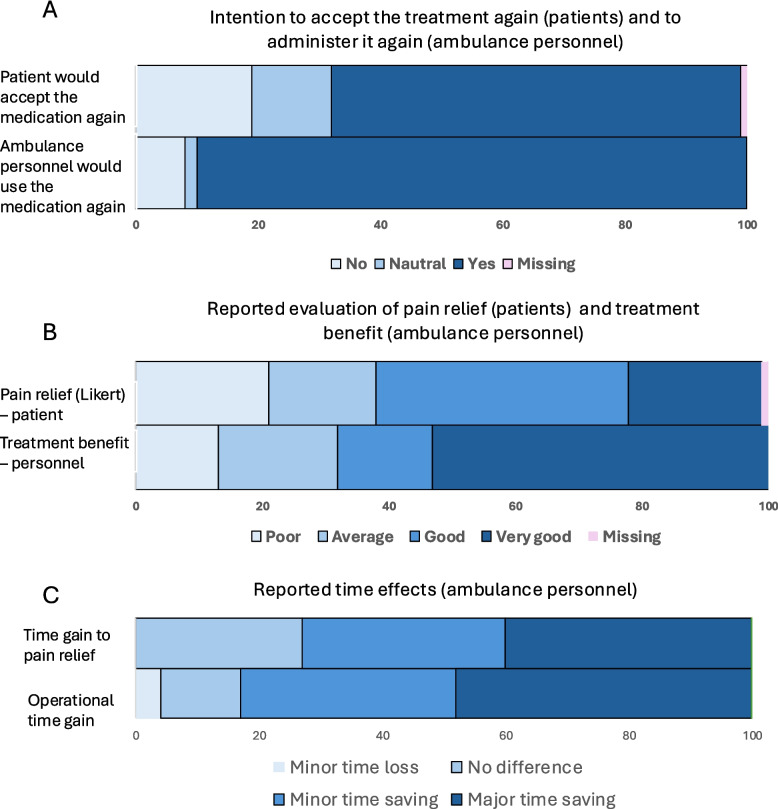


The original article [1] has been corrected.
