# Supplementary material for: MetoksyKval: the extent of pre-hospital methoxyflurane administration for acute traumatic pain: focus on economic impact and rationale for use
Source: Scand J Trauma Resusc Emerg Med. 2026 Jan 9;34:29. doi: 10.1186/s13049-026-01546-z (PMC12882538; doi:10.1186/s13049-026-01546-z)
Supplement: Supplementary file 1 — Additional file 1: Cost overview of analgesics. [file 13049_2026_1546_MOESM1_ESM.pdf]

Additional file 1

Cost overview of analgesics

Item	Unit	NOK*	**Euro**	Source
**Methoxyflurane**				
Methoxyflurane	3 ml dose for vaporisation in a Pentrox® inhaler (Medical Developments NED B.V, Amsterdam, The Netherlands?)	399,00	34,78	1
**Morphine**				
Morphine sulfat	One ampoule contains 1 ml solution 10 mg/ml for injection	42,47	3,70	1
**IV Paracetamol**				
Paracetamol	One vial contains 100 ml solution 10 mg/ml for infusion	17,75	1,55	1
**Esketamine**				
Eskemtamine	One ampoule contains 5 ml solution 5mg/ml for injection.	37,22	3,25	1

*Prices presents are calculated as the average of lower prices for a medication in selected European countries (Sweden, Finland, Denmark, Germany, the United Kingdom, Netherlands, Austria, Belgium, and Ireland).

1: Source Felleskatalogen.no
