# Supplementary figures and images for: MetoksyKval: the extent of pre-hospital methoxyflurane administration for acute traumatic pain: focus on economic impact and rationale for use
Source: Scand J Trauma Resusc Emerg Med. 2026 Jan 9;34:29. doi: 10.1186/s13049-026-01546-z (PMC12882538; doi:10.1186/s13049-026-01546-z)

## Additional file 2

### Location at ambulance station including in the MetoksyKval study

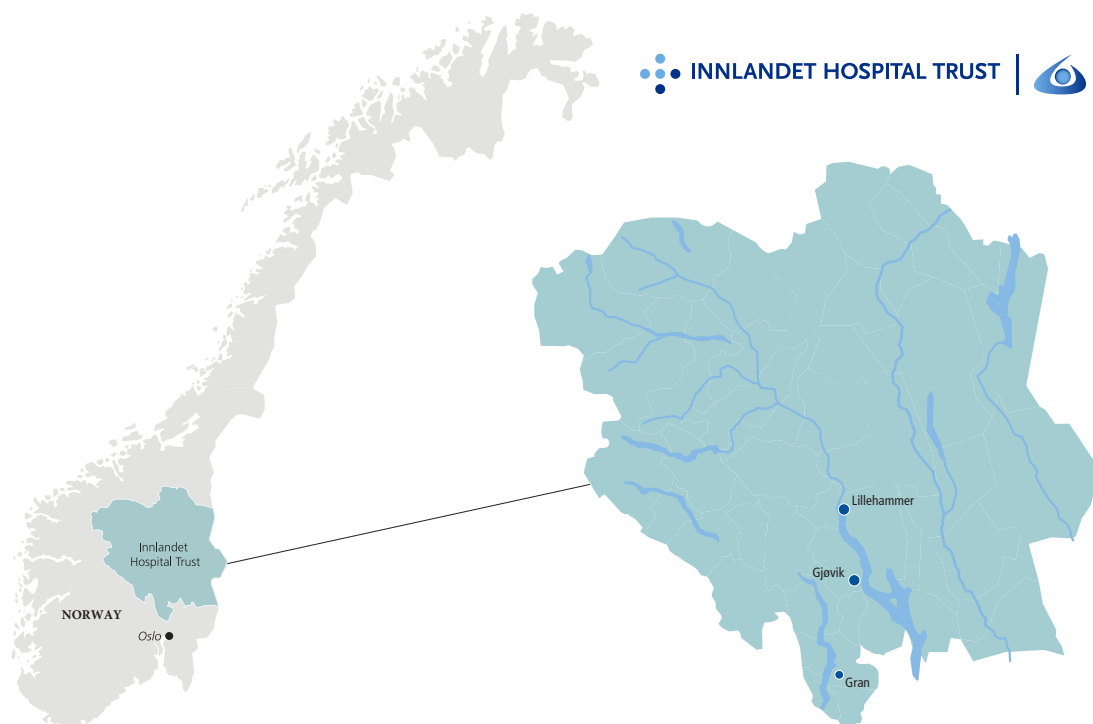

Supplement: Supplementary file 2 — Additional file 2: Location at ambulance station including in the MetoksyKval study. [file 13049_2026_1546_MOESM2_ESM.pdf]
