# Supplementary material for: MetoksyKval: the extent of pre-hospital methoxyflurane administration for acute traumatic pain: focus on economic impact and rationale for use
Source: Scand J Trauma Resusc Emerg Med. 2026 Jan 9;34:29. doi: 10.1186/s13049-026-01546-z (PMC12882538; doi:10.1186/s13049-026-01546-z)
Supplement: Supplementary file 3 — Additional file 3: Calculation: Probability of at Least One Trained Crew Member. [file 13049_2026_1546_MOESM3_ESM.pdf]

Additional file 3

Calculation: Probability of at Least One Trained Crew

Member Total employees = 84

- Trained for study procedure = 51
- Not trained = 84 – 51 = 33
- Each ambulance is staffed by two people, randomly paired

1. **Total number of possible pairs:**

   C(84, 2) = 84 × 83 / 2 = 3486
2. **Number of pairs without trained personnel:**

   C(33, 2) = 33 × 32 / 2 = 528
3. **Number of pairs with at least one trained person:**

   3486 – 528 = 2958
4. **Probability:**

   P(at least one trained) = 2958 / 3486

   = 493 / 581 ≈ 0.8485

Result:
Approximately **84.9%** of ambulance missions will have at least one crew member trained in the procedure on board.
