# Supplementary material for: MetoksyKval: the extent of pre-hospital methoxyflurane administration for acute traumatic pain: focus on economic impact and rationale for use
Source: Scand J Trauma Resusc Emerg Med. 2026 Jan 9;34:29. doi: 10.1186/s13049-026-01546-z (PMC12882538; doi:10.1186/s13049-026-01546-z)
Supplement: Supplementary file 5 — Additional file 5: Inclusion and exclusion criteria. [file 13049_2026_1546_MOESM5_ESM.pdf]

Additional file 5

Inclusion and exclusion criteria

**Inclusion criteria**	**Exclusion criteria**
≥18 years of age	Previous liver damage use of inhaled anesthesia
Moderate to severe pain, resulting from trauma, defined by self-reporting pain ≥4 on Numeric Rating Scale.	Clinically significant renal impairment
Glascow Coma Scale 15	Impaired consciousness (Glasgow Coma Scale <14)
Ambulance personnel consider that methoxyflurane may have an advantage over intravenous analgesia	Cardiovascular instability
	Respiratory depression

BEGRENSET (Unntatt offentligheten)
