# Supplementary material for: MetoksyKval: the extent of pre-hospital methoxyflurane administration for acute traumatic pain: focus on economic impact and rationale for use
Source: Scand J Trauma Resusc Emerg Med. 2026 Jan 9;34:29. doi: 10.1186/s13049-026-01546-z (PMC12882538; doi:10.1186/s13049-026-01546-z)
Supplement: Supplementary file 6 — Additional file 6: Translated version of Consent form. [file 13049_2026_1546_MOESM6_ESM.pdf]

UNIVERSITETET I OSLO NORSK LUFTAMBULANSE STIFTELSEN Sykehuset Innlandet HF Divisjon Prehospitale tjenester

MethoxyQualifier Inclusion and Consent Text

AMIS No.: ___ ___ ___ ___ ___ ___ ___ - ___ **Date:** __ __ /__ __ - 20__

Study work: ______________________________ (personnel number)

Indications for methoxyflurane	YES
NRS ≥ 4	
Over 18 years of age	
Awake and conscious	
Trauma-triggered pain	

→

Check contraindications	
No contraindications	

→ Patient may receive methoxyflura

To be read:

We perform a study on the use of a type of analgesics called Methoxyflurane. The medicine is approved for use for acute pain and is given as an inhalation instead of through a cannula. You will be offered this treatment as a supplement to regular pain treatment, and regardless of whether you accept or decline the inhalation, we will treat your pain so that you feel good.

Information about you and your treatment is collected and stored securely by us. You can withdraw at any time and receive the same good treatment as the usual medications.

Do you agree that we collect information about this processing from you?

End reading

If the patient consents, you as a study worker must tick and sign here:

☐ to have provided information about the study

☐ that the patient has unequivocally consented to the collection and use of personal data as described;

Date:	Signature study work:
	Name (**readable**):
