# Supplementary material for: MetoksyKval: the extent of pre-hospital methoxyflurane administration for acute traumatic pain: focus on economic impact and rationale for use
Source: Scand J Trauma Resusc Emerg Med. 2026 Jan 9;34:29. doi: 10.1186/s13049-026-01546-z (PMC12882538; doi:10.1186/s13049-026-01546-z)
Supplement: Supplementary file 7 — Additional file 7: Additional treatment per Innlandet Hospital Trust analgesic protocols. [file 13049_2026_1546_MOESM7_ESM.pdf]

Additional file 7

Additional treatment per Innlandet Hospital Trust analgesic protocols

Administration method	Name	ATC code
IV/PO/SUPP	Paracetamol	N02 B E01
IV/IN/IM	Ketamine	N01 A X03
IV/IN/IM	Esketamine	N01 A X14
IV/IM	Morphine	N02 A A01
IN	Fentanyl	N02 A B03
IM/PO	Diclofenac	M01A B05

Legends: Includes all analgesics other than the study drug. Additional treatment was given only if the study drug was insufficient, selected from analgesics available in the ambulance and dosed per local procedures Abbreviations : IV=intravenous; IM=intramuscular; IN=intranasal; PO=per oral; SUPP= Suppository ; ATC= Anatomical Therapeutic Chemical
