# Supplementary material for: MetoksyKval: the extent of pre-hospital methoxyflurane administration for acute traumatic pain: focus on economic impact and rationale for use
Source: Scand J Trauma Resusc Emerg Med. 2026 Jan 9;34:29. doi: 10.1186/s13049-026-01546-z (PMC12882538; doi:10.1186/s13049-026-01546-z)
Supplement: Supplementary file 8 — Additional file 8: Numeric Rating Scale of pain with missing values. [file 13049_2026_1546_MOESM8_ESM.pdf]

Additional file 8

Numeric Rating Scale of pain with missing values

	Median	IQR		Missing, n (%)	Analysis denominator
Baseline*	8	6	9.5	0 (0)	48
5 minutes	5	4	8	1 (2)	47
10 minutes	5	4	7	4 (8)	44
Handover	4	3	6	11 (23)	37

Abbreviations: NRS= numeric rating Scale; IQR = interquartile Range. Missing describes values not documented in the case report file. *Baseline measured before treatment.
