# Supplementary material for: MetoksyKval: the extent of pre-hospital methoxyflurane administration for acute traumatic pain: focus on economic impact and rationale for use
Source: Scand J Trauma Resusc Emerg Med. 2026 Jan 9;34:29. doi: 10.1186/s13049-026-01546-z (PMC12882538; doi:10.1186/s13049-026-01546-z)
Supplement: Supplementary file 9 — Additional file 9: Intravenous access and reported reasons for omission. [file 13049_2026_1546_MOESM9_ESM.pdf]

Additional file 9

Intravenous access and reported reasons for omission

	Number of patients	% of N=48
Patients with intravenous access	29	60 %
Patient without intravenous access	17	34 %
**Reason why intravenous access was not established**		**% of N=48**
Cannulation failure	7	15%
Not require intravenous access	6	13 %
Refused Intravenous intervention	4	8%
Miscellaneous	2	4 %

Legdens: Overview of patients with and without intravenous access, and reasons reported for cases where cannulation was not established.
